# RNAi combining *Sleeping Beauty* transposon system inhibits *ex vivo* expression of foot-and-mouth disease virus VP1 in transgenic sheep cells

**DOI:** 10.1038/s41598-017-09302-1

**Published:** 2017-08-30

**Authors:** Shoulong Deng, Guangdong Li, Kun Yu, Xiuzhi Tian, Feng Wang, Wenting Li, Wuqi Jiang, Pengyun Ji, Hongbing Han, Juncai Fu, Xiaosheng Zhang, Jinlong Zhang, Yixun Liu, Zhengxing Lian, Guoshi Liu

**Affiliations:** 1 Beijing Key Laboratory for Animal Genetic Improvement, National Engineering Laboratory for Animal Breeding, Key Laboratory of Animal Genetics and Breeding of the Ministry of Agriculture, College of Animal Science and Technology, China Agricultural University, Beijing, China; 2 State Key Laboratory of Stem Cell and Reproductive Biology, Institute of Zoology, Chinese Academy of Sciences, Beijing, China; 3 Tianjin Institute of Animal Sciences, Tianjin, China

## Abstract

Foot and mouth disease, which is induced by the foot and mouth disease virus (FMDV), takes its toll on the cloven-hoofed domestic animals. The *VP1* gene in FMDV genome encodes the viral capsid, a vital element for FMDV replication. *Sleeping Beauty (SB)* is an active DNA-transposon system responsible for genetic transformation and insertional mutagenesis in vertebrates. In this study, a conserved *VP1*-shRNA which specifically targets the ovine FMDV-*VP1* gene was constructed and combined with *SB* transposase and transposon. Then, they were microinjected into pronuclear embryos to breed transgenic sheep. Ninety-two lambs were born and the *VP1*-shRNA was positively integrated into eight of them. The rate of transgenic sheep production in *SB* transposon system was significantly higher than that in controls (13.04% *vs*. 3.57% and 7.14%, *P* < 0.05). The ear fibroblasts of the transgenic lambs transfected with the PsiCheck2-*VP1* vector had a significant inhibitory effect on the *VP1* gene of the FMDV. In conclusion, the *VP1*-shRNA transgenic sheep were successfully generated by the current new method. The ear fibroblasts from these transgenic sheep possess a great resistance to FMDV. The result indicated that RNAi technology combining the “*Sleeping Beauty*” transposon system is an efficient method to produce transgenic animals.

## Introduction

Foot and mouth disease (FMD) is an acute, febrifacient and highly contagious disease to the mammals which are susceptible for them. It easily and rapidly spreads through the air and causes enormous economic losses^1^. The culprit responsible for this disease is the foot and mouth disease virus (FMDV), which is a picornavirus and belongs to a prototypical member of the Aphthovirus genus. The virus particle (25–30 nm), which has no envelope, has an icosahedral capsid, a protein containing a single strand of ribonucleic acid (RNA), which positively encodes its genome. When the virus encounters the membrane of a host cell, it binds to a receptor site and triggers an infolding of the membrane. Once the virus enters the host cell, the capsid dissolves, and the RNA is replicated and translated into viral proteins by the cell ribosomes using a cap-independent mechanism driven by the internal ribosome entry site element. The FMDV genome consists of a 5′ untranslated region (5′-UTR), an open reading frame (ORF), the 3′-UTR and poly-A tails^2^. The P1 region in the ORF encodes four structural proteins called VP1, VP2, VP3 and VP4, respectively, which form the viral capsid^3^. In particular, VP1 is the pivotal antigenic variation site, most of which is exposed to the virus protein surface that mediates viral infection of cells. In FMD molecular epidemiology research, the *VP1* gene nucleotide sequence is often determined and analyzed^4^.

RNA interference (RNAi) is an RNA-dependent gene-silencing process controlled by the RNA-induced silencing complex (RISC), and it is initiated by short, double-stranded RNA molecules in the cytoplasm of a cell that interact with the catalytic RISC component argonaute^5^. RNAi is highly effective to inhibit *VP1* replication in transient assays with small interfering RNA (siRNA)^6^, and more durable inhibition can be achieved when an antiviral short hairpin RNA (shRNA) is expressed in stably transfected or transduced cell lines^7, 8^. shRNAs, different from siRNAs, are synthesized in the cell nucleus, further processed and transported to the cytoplasm, and then incorporated into the RISC, where they become active^9^. They can be transcribed by either RNA polymerase II or III through RNA polymerase II or III promoters on the expression cassette^10^. Owing to the stability of shRNA, it is increasingly being used to develop antisense therapeutics, that is post-transcriptionally knockdown gene expression^11^. Compared to traditional vaccines, the RNAi approach is preferred for the inhibition of FMDV infection and replication due to its direct effect on the FMDV genome. By targeting *VP1* gene-conserved sequences, previous studies have designed one shRNA that can effectively control FMDV infection by inhibiting *VP1* gene duplication and further silencing the expression of this protein^6^.

The *SB* transposon system is a synthetic DNA transposon designed to introduce precisely defined DNA sequences into the chromosomes of vertebrates to introduce new traits as well as to discover new genes and their functions. It consists of two components: the transposon and the transposase. The transposon is the DNA surrounded by the two-terminal inverted repeat (IR)/direct repeat (DR) elements, and the transposase is the protein that facilitates transposition by binding to the DR regions within the IR/DR elements. Together, these two components act in a cut-and-paste manner to move the entire transposon from the donor plasmid or location to a thymine-adenine (TA) dinucleotide within the recipient DNA fragment^12^. Previous reports have described an integrated transposon system in which the transposon and transposase are built in separate expression vectors so that other DNA fragments can translocate to the primary transposase sites^13^. With this system, *SB* transposons can carry any foreign DNA fragments and integrate them into animal genomes to achieve stable transposon-mediated insertional mutagenesis.

In 2015, we successfully bred sixty-one goats, of which seven individuals positively integrated *3D*-7414shRNA targeting the *3Dpol* gene (which encodes the viral RNA polymerase, a vital element for FMDV replication) in the FMDV genome^14^. However, few studies have integrated the *SB* transposon system with RNAi technology in farm animals, so this study investigated the prospect of producing anti-FMD sheep by utilizing *VP1*-shRNA coupled with the *SB* transposon system.

## Results

### VP1-shRNA inhibits the expression of FMDV-VP1

**Figure 1:**
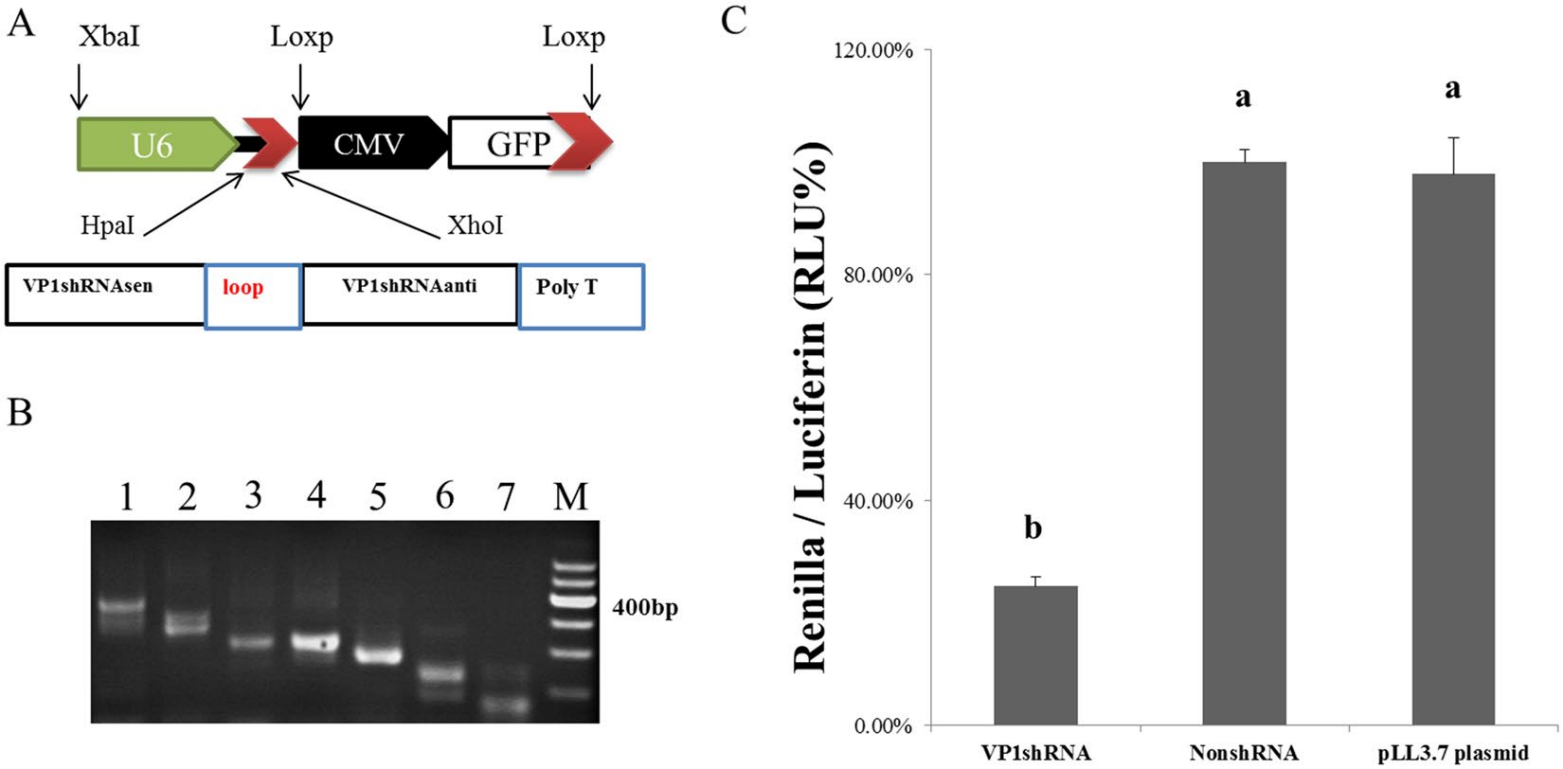
Inhibitory effects of *VP1*-shRNA on FMDV-*VP1*. (**A**) Schematic map of the pLL3.7-*VP1*-shRNA expression vector. (**B**) The *VP1* gene amplification using overlapping PCR; 1: a-h, 2: a-g, 3: a-f, 4: a-e, 5: a-d, 6: a-c, 7: a-b, and M: marker. (**C**) Use of Dual-Glo luciferase to detect the inhibitory effect of targeted genes. 239FT cells were co-transfected with pll3.7-shRNA and psiCheck2 genes with different ratios, and the expression of Dual-Glo luciferase reporter genes was measured after 48 h. Data were expressed as the means ± S.E.M. (n = 3). Columns with different superscripts differ significantly, *P* < 0.05.

The FMDV-*VP1* sequences were analyzed, and the shRNA sequences were screened and synthesized. After annealing, the sense and antisense strands were cloned into the pLL3.7 vector (Fig. 1A), The *VP1* gene was obtained by overlapping PCR (Fig. 1B) and cloned into vector psiCheck2, resulting in a new vector psiCheck2-*VP1*, which was co-transfected with pLL3.7-NonshRNA or pLL3.7-*VP1*-shRNA, respectively (4:1), into 293FT. The Dual-Glo Luciferase Assay System was used to explore the activities of firefly luciferase and renilla luciferase. The formula [(renilla/firefly luciferase activity)_empty group_ − (renilla/firefly luciferase activity)_shRNA group_]/(renilla/firefly luciferase activity)_empty group_ accounts for the inhibition rate. The results indicated that, the inhibition efficiency of *VP1*-shRNA was 75.22% higher than that non-shRNA (*P* < 0.05) (Fig. 1C).

### Production of transgenic sheep through the SB transposon system

**Figure 2:**
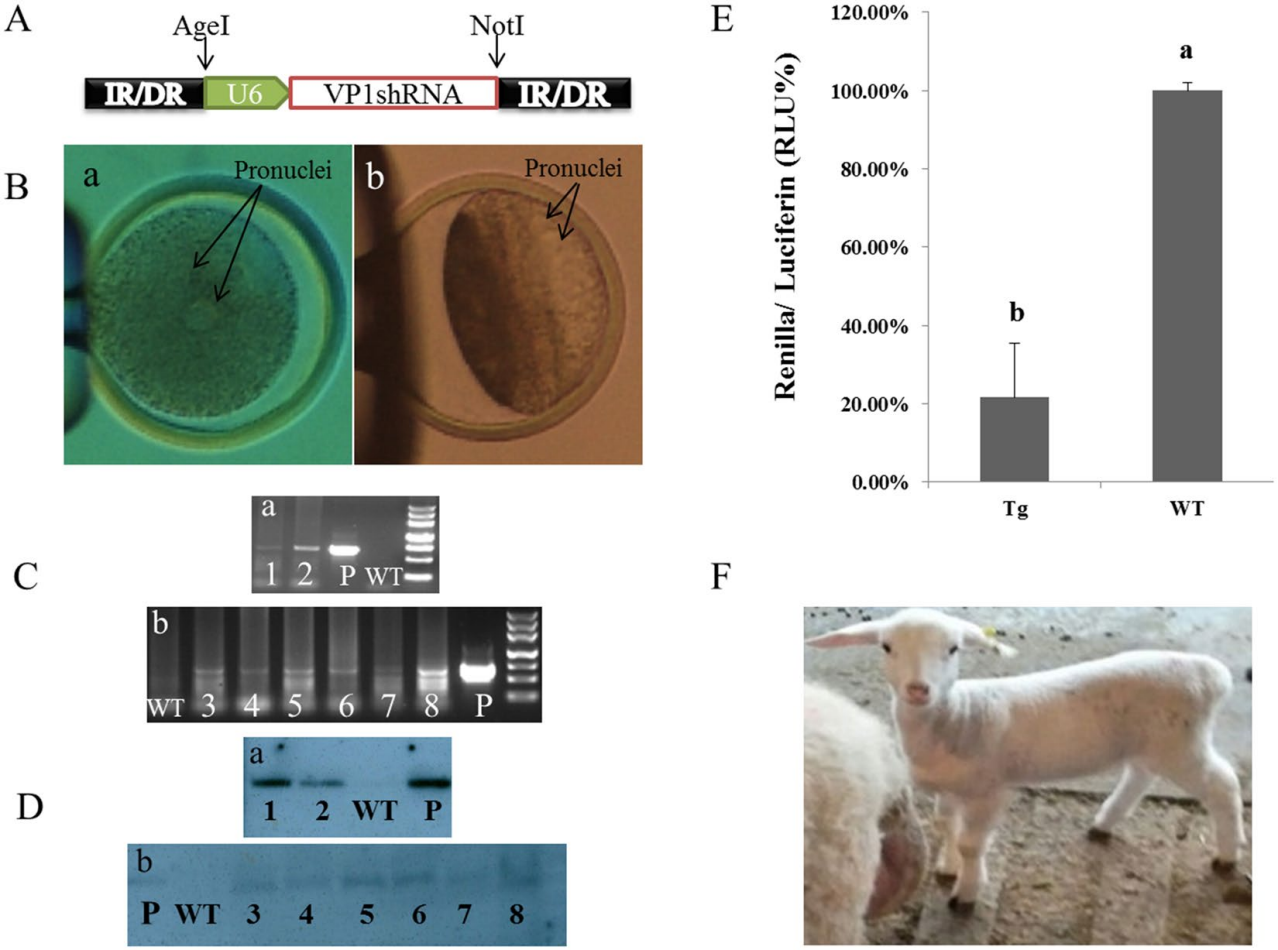
Identification of *SB* transposon-mediated transgenic sheep. (**A**) Schematic diagram of the inserted part of the pUC-*VP1*-shRNA expression vector, which was cut from pLL3.7 and ligated into the PUC19-IR/DR vector. (**B**) Pronuclear microinjection. (a): non-centrifuged fertilized ovine egg and (b): centrifuged fertilized egg (12000 × g for 5 min). (**C**) Electrophoresis of PCR products and (**D**) Southern blot analysis to identify the transgenic sheep, respectively. a: U6-*VP1*-shRNA group and linearized pLL3.7-*VP1*-shRNA plasmid group. b: IR/DR-U6-*VP1*-shRNA + *SB100* × group. WT = wild type as a negative control; “p”: positive control; “1–8”: transgenic lambs. (**E**) Inhibitory effects of shRNA on *VP1* gene expression in ear fibroblasts of transgenic versus wild type sheep as determined by luciferase reporter assay. Each test was repeated three times for each individual. Tg ( = transgenic sheep), N = 8; WT (wild type), N = 8. (**F**) A photo of the transgenic lamb. Data were expressed as the means ± S.E.M. Columns with different superscripts differed significantly, *P* < 0.05.

**Figure 3:**
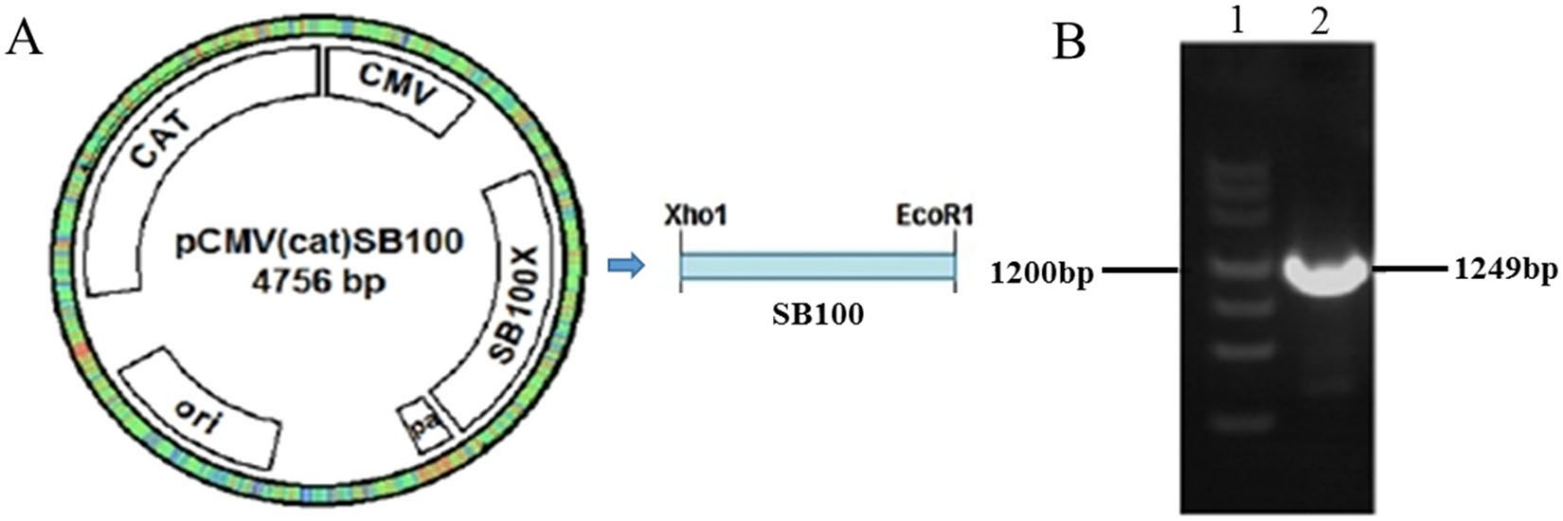
Identification of the *SB* transposase gene. (**A**) A diagraph of construction of the *SB* transposase gene (**B**) The PCR result of *SB* transposase gene *SB100* × based on the template pCMV*SB100* vector. 1: Marker, 2: *SB100* × fragment.

The *SB* transposon expression vector pUC-*VP1*-shRNA was constructed (Fig. 2A) and was successfully integrated into eight transgenic sheep (Fig. 2F) by pronuclear microinjection (Fig. 2B). Southern blot analysis was used to detect the positive transgenic lambs. By analyzing the developed blots to calculate the transgene copy numbers, in which the individuals were identified to carry various copy numbers of the exogenous gene. In the U6*-VP1*-shRNA group, 160 fertilized eggs were transplanted into 43 recipients and 28 lambs were obtained. However, PCR and Southern blot analysis showed that only one of them was transgenic positive (Figs. 2C-a-1,D-a-1). In the linearized pLL3.7-*VP1*-shRNA group, 63 fertilized eggs were transplanted into 14 recipients and 14 lambs were obtained and only one of them was transgenic positive (Fig. 2C-a-,D-a-2). In the linearized IR/DR-U6-*VP1*-shRNA + *SB100* × group, 153 fertilized eggs were transplanted into 40 recipients and 23 lambs were obtained and 3 of them were transgenic positive (Figs 2C-b-3,4,5;D-b-3,4,5). The numbers of exogenous gene copies were calculated, which were 4.38, 2.13, 1.21, 1.19 and 1.82, respectively (Fig. 2D:1–5). The efficiency of producing transgenic sheep with the *SB* transposon system was 13.04%, which is higher than that of the U6-*VP1*-shRNA group and the linearized pLL3.7-*VP1*-shRNA group (*P* < 0.05) (Table 1).

**Table 1:** Efficiency of transgenic sheep production by pronuclear microinjection.

Gene	No. of ET	Recipients	Survival	Positive rate (%)
U6-*VP1*-shRNA	160	43	28	3.57 (1/28)^c^
pLL3.7-*VP1*-shRNA	63	14	14	7.14 (1/14)^b^
IR/DR-U6-*VP1*-shRNA:*SB100*×	153	40	23	13.04 (3/23)^a^

The data were expressed as means ± SEM. Values with different superscript letters represent a significant difference between different groups, (P < 0.05).

In addition, the pronuclei, in IR/DR-U6-*VP1*-shRNA + *SB100* × group, were better visualized than that in other groups.,In the current study, some fertilized eggs of this group were centrifuged. It was found that after centrifugation, the lipid droplets in the cytoplasm were gathered to one side (Fig. 2B-b). In the centrifuged group, 132 fertilized eggs were transplanted into 33 recipients and 14 lambs were obtained. PCR and Southern blot analysis showed that one of them was transgenic positive (Fig. 2C-b-6,D-b-6); the positive rate of the centrifuged group was 7.14%. However, in the un-centrifuged group, 63 fertilized eggs were transplanted into 21 recipient and 13 lambs were obtained, two of them were transgenic positive (Fig. 2C-b-7,8, D-b-7,8), the positive rate of the un-centrifuged group was 15.38%, higher than that in centrifuged group (*P* < 0.05) (Table 2). The numbers of exogenous gene copies were calculated, which were 1.79, 1.12 and 1.92, respectively (Fig. 2D:6–8).

**Table 2:** Efficiency of transgenic sheep production by centrifugation relative to the control.

Group	No. of ET	Recipients	Survival	Positive rate (%)
Centrifuged	132	33	14	7.14 (1/14)^b^
Control	63	21	13	15.38 (2/13)^a^

The data were expressed as means ± SEM. Values with different superscript letters represent a significant difference between different groups, (P < 0.05).

In the IR/DR-U6-VP1-shRNA + *SB100* × group, to study the effect of different ratio of transposon and transposase on the transgenic efficiency, variable concentrations of transposon and transposase were microinjected into ovine zygotes. It was found when the ratio of transposon to transposase was 1:5, the pregnancy rate was 42.42%, and the PCR positive rate of the progeny was 19.05%. However, when this ratio was 5:1, the pregnancy rate was 26.14%, and the PCR positive rate of the progeny was 35.48%. When the ratio was 10:1, the pregnancy rate was 30.33%, and the PCR positive rate of the progeny was 33.33% (Table 3).

**Table 3:** Effects of different transposon and transposase ratios on the transgenic efficiency of ovine pronuclear microinjection.

Ratio	Dose	Pregnancy rate (%)	PCR positive rate (%)
IR/DR-U6-*VP1*-shRNA*:SB*100 × 1:5	5~10 ng/μl	42.42% (14/33)	19.05 (4/21)^b^
IR/DR-U6-*VP1*-shRNA:*SB*100 × 5:1	5~10 ng/μl	26.14% (23/88)	35.48 (11/31)^a^
IR/DR-U6-*VP1*-shRNA:*SB*100 × 10:1	5~10 ng/μl	30.33% (27/89)	33.33 (11/33)^a^

The data were expressed as means ± SEM. Values with different superscript letters represent a significant difference between different groups, (P < 0.05); The same letters represent no significant difference (P > 0.05).

### Fibroblasts of transgenic sheep ear tissue inhibit VP1 gene expression

To compare the inhibitory effects of transgenic animals on VIP gene expression with non-transgenic ones (each group has 8 lambs), we transfected the psiCheck2-VP1 into the ear fibroblast cells derived from both group. The inhibition rate was determined by the activities of firefly luciferase and renilla luciferase, which were measured through the Dual-Glo Luciferase Assay System. The results showed that the cells from transgenic animals significantly inhibited the expression of *VP1* gene compared with the wild type (*P* < 0.05) (Fig. 2E).

## Discussion

FMDV causes an acute vesicular disease in livestock, and vaccination is currently the main preventive method^15, 16^. To a certain extent, vaccinated animals are resistant to FMDV, but there are many drawbacks, such as the short-lived effectiveness of the resulting antibodies^17^. In contrast, RNAi is an ideal method for inhibiting FMDV infection and replication because it can directly affect the FMDV genome^18, 19^. The FMDV genome encodes many genes, including *2B*, *3Dpol*, *VP1*, *VP2*, *VP3*, *VP4*, *VPg*, *etc*. These genes have been selected as RNAi targets^20–23^. *VP1* is essential during the life cycle of the virus and plays a key role in its attachment to susceptible cells^24, 25^. In this study, we screened and constructed the valid shRNA recombinant vectors (Fig. 1A) targeting the conserved region of *VP1* to inhibit its replication and further silence the expression of FMDV. The anti-FMDV shRNA proved to be effective with an inhibition efficiency of 75.22% in 293FT cells compared with Non-shRNA cells (*P* < 0.05) (Fig. 1C). Furthermore, ear fibroblast cells from the positively integrated *VP1*-shRNA sheep also significantly inhibited the expression of *VP1* gene compared with wild type animals (*P* < 0.05) (Fig. 2E). In transgenic bovines, it has been reported that the primary epithelium cells with shRNA expressing RNAi-LT4 (VP4) or RNAi-LT6 (VP1) showed greater resistance to FMDV than those in non-transgenic cells^20, 26^, which is similar to our findings.

There are currently many methods for producing transgenic animals, such as pronuclear microinjection, retrovirus infection, somatic cell cloning, etc.; however, these transgenic technologies are still to be improved to increase their efficiency. *SB* was the first transposon shown to be capable of efficient transposition in vertebrate cells, thus enabling new avenues for genetic engineering in animal models^27^ that can mediate the stable integration and long-term expression of foreign genes^28^. *SB* transposon vectors have been shown to efficiently deliver a wide variety of transgene cassettes^29^, including shRNA expression cassettes to obtain stable RNAi knockdown cell lines as well as cassettes inducing gain-of-function and loss-of-function gene mutations^30^. The integrated *SB* transposon has been shown to be stably transmitted to offspring through germ cells and maintained for several generations in all species tested^31^. Compared with the viral vector, the *SB* transposon system has many advantages, such as a simple structure, a less-restricted inserted fragment length, controllable exogenous gene expression and higher transgenic efficiency. Combining RNAi-mediated gene silencing and *SB* transposition has been used to down-regulate gene expression, and examples include *SB*-mediated RNAi targeting the lamin A gene^32^, the multidrug-resistance gene *MDR-1* ^33^, the HIV-1 co-receptor genes *CCR5* and *CXCR4* ^34^, and the huntingtin gene *Htt* ^35^, etc. However, previous studies have mainly focused on the cellular level rather than the individual level. Here, we developed a transposon-based vector system that allows the stable expression of shRNA through the microinjection of pronuclear embryos to produce genetically modified ovine species as well as to achieve disease resistance through animal breeding. Thus, a stable knockdown expression system, such as that described here, can overcome the obstacle of transient knockdown of durable proteins by enabling long-lasting production of siRNA oligonucleotides. Our results indicate that the non-viral plasmid-based approach to *VP1*-shRNA cassette delivery is more efficient with the *SB* transposon system than without it as to generate transgenic animals, The transgenic positive offspring with *SB* system was 13.04% (3/23) compared to the others without *SB* system 3.57% (1/28) and 7.14% (1/14), respectively, *P* < 0.05 (Table 1). The transgenic positive rate was also higher than that previously reported^36^.

The efficiency of the *SB* transposon system is affected by many factors including species, cell lines, transposon length, copy number, lateral sequences, transposase, transposon to transposase ratio, etc.^37^. Zsuzsanna Izsvák *et al*. transfected the *SB* transposon system into the A6 cell line of Xenopus followed by screening with neomycin and found the efficiency to be only 0.03%, but an 8% transposition rate was reported in the hamster K1 cell^38^. The authors also showed that transposition efficiency would be reduced by 30% per additional 1 Kb *SB* transposons in Hela cells^38^. Yant *et al*. reported when the ratio of the transposon plasmid to the transposase was 25:1 the highest transposition efficiency was achieved during the transfection of mouse liver cells^39^, but Geurts *et al*. found that the highest transposon efficiency was when the transposon to transposase ratio was 5:1 in Hela cell transfection^40^. In the current study, 35.48% transposon efficiency was achieved when the transposon to transposase ratio was 5:1, which was higher than other ratios of 1:5 or 10:1 (19.05% and 33.33%, respectively, *P* < 0.05) (Table 3); these results were consistent with those of Geurts *et al*. but differed from Yant *et al*. These discrepancies may be explained by species-specific differences in the integration and viability of embryos, gene constructs and microinjection facilities.

Pronuclear embryo microinjection technology involves the injection of exogenous genes into the pronuclei of fertilized eggs using a micromanipulator. The pronuclei of late zygotes are located close to one another, usually in the center of the cytoplasm although they may be closer to the pole with dark granules; one of the pronuclei (male) is somewhat larger than the other (female). Pronuclei in sheep, unlike those in mouse, rabbit, or swine, are not visualized, and they morphologically resemble goat and cow pronuclei^41^. The two pronuclei may not always be simultaneously visible, and one can be located in the lipid granules. Nontransparent cytoplasm is due to the presence of a large amount of lipid granules, which hinders pronuclei visualization and affects microinjection^42^. However, previous reports have mainly focused on the pronuclei of goats rather than sheep; for example, Freitas *et al*.^42^ centrifuged fertilized, one-cell goat embryos at 13400 rpm for 4–6 min to visualize the pronuclei rather than 15600 × g for 3–4 min as described by Baldassarre *et al*.^41^. In this study, we conducted pronuclear microinjection by centrifuging some fertilized eggs at 12000 × g for 5 min (Fig. 2B-b) to try to visualize the pronuclei better. In the rest of the fertilized eggs, we were unable to observe the morphology of the pronuclei in detail, thus, the microinjections were performed at the most likely location of the pronuclei. In these cases, it was difficult to control the quality of the injected eggs. For example, by visualizing whether there were significant change in pronucleus morphology due to swelling or other reasons. Even through, the proportion of clearly visible pronuclei increased with centrifugation, the efficiency of transgenic sheep production is reduced (Table 2), presumably due to skeletal damage to the fertilized egg after centrifugation.

## Conclusions

In summary, the combination of *Sleeping Beauty* transposon expression vector with RNAi and pronuclear microinjection technology significantly increased the genetic positive offspring in sheep. By use of this combination, 8 transgenic sheep expressing *VP1*-shRNA targeting the *VP1* gene in the FMDV genome were generated out of 92 individuals. In addition, we proved that the ear fibroblasts from transgenic lambs could significantly inhibit VP1 replication compared to the cells from the wild type. Moreover, it was found that *SB* transposon-mediated shRNA had an increased transgenic integration rate over random integration of anti-*VP1* shRNA. The DNA-mediated transposition may suggest new ways to further improve this novel integration system.

## Materials and Methods

### Chemicals

Unless specified, reagents were purchased from Sigma Chemical Co. (St. Louis, MO, USA).

### Animal studies and ethics statement

All transgenic sheep production and sample collection procedures were strictly followed the protocols approved by the Animal Welfare Committee of China Agricultural University (Permit Number: XK662), and this study was carried out in strict accordance with the guidelines and regulations established by this committee. The experimental ewes were aged 1 to 4 years old and were free of reproductive diseases. They were sufficiently fed to achieve good condition at least one month prior to the experiment. The experimental rams weighed 75~85 kg and had a good sex drive and high-quality semen.

### Vector construction

Target screening and construction of shRNA recombinant vectors were performed as follows. One candidate shRNA, *VP1*-shRNA, targeting FMDV (GenBank accession: AY686687.1) was designed with BLOCK-iTRNAi Designer (http://rnaidesigner.lifetechnologies.com/rnaiexpress), and the sequences were as follows: *VP1*-shRNAsen (5′-TGGAGTCTGCGGACCCCGTGACT- TTCAAGAGAAGTCACGGGGTCCGCAGACTCCTTTTTTC-3′), *VP1*-shRNAanti (5′-TCGAG- AAAAAAGGAGTCTGCGGACCCCGTGACTTCTCTTGAAAGTCACGGGGTCCGCAGACT-CCA-3′), Non-shRNA-sense (5′-TGGCAGTACGGAACTCCGCTAGGTTCAAGAGACCTAG -CGGAGTTCCGTACTGCCTTTTTTC-3′), and Non-shRNA-anti (5′-TCGAGAAAAAAGGCA- GTACGGAACTCCGCTAGGTCTCTTGAACCTAGCGGAGTTCCGTACTGCCA-3′). The sequences were synthesized by Shanghai Sangon Biological Engineering Technology and Service Co., Ltd. (Sangon, China). The two shRNAs were cloned into the pLL3.7 expression vector (Addgene, USA) to obtain pLL3.7-*VP1*-shRNA and pLL3.7-NonshRNA, which were located after promoter U6, and *HpaI* and *XbaI* were the restriction enzyme cutting sites. The construction of the shRNA vector in the *SB* transposon system was as follows. The IR/DR sequences of the pT2 vector (Sino-US Ltd.) were inserted into the *Sma I* sites of the pUC19 vector (Addgene, USA), namely, PUC19-IR/DR. The U6-*VP1*-shRNA, which was cut by *Not I* and *XbaI* in pLL3.7, was ligated into the IR/DR of the PUC19-IR/DR vector, resulting in a new vector named pUC-*VP1*-shRNA. The *SB* transposase gene *SB100* × (Forward primer 5′-ATGGGAAAA- TCAAAAGAAATCAGC-3′ and Reverse primer 5′-CTAGTATTTGGTAGCATTGCC-3′ with a product size of 1249 bp) was amplified by PCR based on the pCMV*SB100* vector template (Fig. 3). The *SB100* × mRNA was obtained by *in vitro* transcription using mMessage mMACHINE T7 Ultra Kit (Ambion, AM1345) according to the manufacturer’s protocol.

### Analysis of shRNA inhibition efficiency

The *VP1* gene was chemically synthesized by designing 8 primers (Table 4) based on the FMDV-conserved sequence and cloned into the psiCheck2 plasmid (Promega, USA) between *Xho I* and *Not I* to obtain psiCheck2-*VP1*. Then, psiCheck2-*VP1* was co-transfected with pLL3.7-NonshRNA or pLL3.7-*VP1*-shRNA, respectively (4:1), into 293FT by Lipofectamine 2000 (Invitrogen, USA); the cells were transfected with pLL3.7 plasmid as an empty control. The 293FT cells were grown in Dulbecco’s modified Eagle’s medium (DMEM; Invitrogen, USA) supplemented with 10% fetal bovine serum (FBS; Gibco, USA). All transfection experiments were performed in triplicate. The activities of firefly luciferase and renilla luciferase were detected by the Dual-Glo Luciferase Assay System (Promega, USA) at 48 h post transfection, and the inhibition rate was calculated by the formula [(renilla/firefly luciferase activity)_empty group_ − (renilla/firefly luciferase activity)_shRNA group_]/(renilla/firefly luciferase activity)_empty group_.

**Table 4:** The *VP1* gene primers.

*VP1* gene	Sequence (5′–3′)
a	ACCTCGAGTGCGGGTGAGTCTGCGGACCCCGTGACTACCACCGTCGAAAACTACGGCGG
b	TGAACGCAACGTCCGTGTGTTGGCGCCTCTGGACTTGTGTCTCGCCGCCGTAGTTTTCG
c	GTTAACTTGCTCCTGTGGTTTGACTTTCACGAACCTGTCCAATATGAACGCAACGTCCG
d	GCCCCTACCAAGGTGTGGGCAGGGATCTGCATCAGGTCCAACACGTTAACTTGCTCCTG
e	CTAGTTCCAGGTCAGAGAAGTAATAGGTGGCCGTCCGCAGGAGTGCCCCTACCAAGGTG
f	GGCACCGTTTGGAACCCAGGTGAGATCGCCCTCGTGCTTGACAGCTAGTTCCAGGTCAG
g	TGGTAGGCTGTTGGGTTGGTGGTGTTGTTCAGTGCTGCCTCGGGGGCACCGTTTGGAAC
h	ATCTCGAGGTATAAGGCAGCGCCAGCCGTGTGAGCGGTTCCTTGTGGTAGGCTGTTGGG

### Superovulation and artificial insemination of donor sheep

The estrous cycles of the ewes were synchronized by a 14 day treatment using a controlled internal drug release device (CIDR; EAZI-BREED® CIDR® Sheep and Goat Device, Pfizer Animal Health, New Zealand) containing 300 mg of progesterone. The CIDR was placed into the vagina of the donor sheep during their estrous cycles, and after 14 days, follicle stimulating hormone (FSH) (OVAGENTM, ICP, Auckland, New Zealand) was injected for superovulation. The donor sheep were in their estrus 24~48 h following the terminal FSH injection. Outstanding rams were selected as sperm donors. Their semen was diluted by diluent and examined for motility; sperm was only used for artificial insemination if the motility reached 0.6. Insemination was timed based on follicle development, which was determined by abdominal endoscopy (Karl Storz Endoskope GmbH, Tuttlingen, Germany); the infusion dose was 0.3 mL. The CIDR + FSH protocol followed that of previous studies with modifications^43^.

### Acquisition of pronuclei and gene microinjection

All sheep were deprived of food for 12 h before embryo recovery to facilitate surgery and to reduce post-operation intestinal adhesions. The pronuclei, which were flushed from the oviduct with a flushing medium (PBS + 0.3% BSA) (Fraktion, 735078; Roche Diagnostics, GmbH, Mannheim, Germany) by peritoneal operation following 18 h of insemination, were aspirated with a micropipette and placed in a small petri dish containing a holding medium (Immuno-Chemical Products Ltd., Auckland, New Zealand) enriched with 20% serum for use after being examined for quality on a thermal plate at 38 °C under a stereomicroscope (SZ61; Olympus, Kawasaki, Japan). The acquired pronuclei were immediately microinjected and then cultured *in vitro* at 38.5 °C in a chamber with 5% CO_2_ and a humidified atmosphere for 30 min. Microinjection was performed with an inverted microscope equipped with DIC and a pair of micromanipulators (Narishige, Tokyo, Japan). The DNA to be injected was divided into 3 groups: the U6-*VP1*-shRNA group (cut from the pLL3.7-*VP1*-shRNA plasmid), the linearized pLL3.7-*VP1*-shRNA plasmid group (linearization by *AgeI* digestion), and the IR/DR-U6-*VP1*-shRNA + *SB100* × group (IR/DR-U6-*VP1*-shRNA cut from pUC19-*VP1*-shRNA and mixed with *SB100* × mRNA at a 5:1 ratio). To facilitate clearness of the pronuclei and promote precipitation of the lipid granules, the centrifuged group (12000 × g for 5 min)^44^ and un-centrifuged control groups were subset among the IR/DR-U6-*VP1*-shRNA + *SB100* × groups. The final concentration and dose of the DNA or mRNA were 5 ng/μL and 5 pL, respectively. The gene was injected into each embryo as described previously^45^.

### Recipient preparation and embryo transfer

The FSH + CIDR donor synchronization method was used; while injecting prostaglandin (PG) (Sansheng Pharmaceuticals, Ningbo, China) 280 IU, the CIDR was removed half a day before the donor. Twelve hours after injection, the ram test was conducted 3 times a day to confirm that the recipient ewe was in estrus; the surgical site and preparation methods for the recipient were consistent with those of the donors. Whether the corpus luteum of the recipient could be transplanted was determined by peritoneal operation through a 2~3 cm cavity in the abdomen. Approximately 2 to 6 embryos were transplanted into the uterine horn per recipient as previously described^46^, and early pregnancy was diagnosed by an ultrasonic instrument (Vetko Plus, Noveko, Quebec, Canada) 50 days later.

### Identification of the DNA level in transgenic sheep

The genomic DNA of the lambs was extracted and analyzed by PCR and Southern blotting as described by Annemarie Hübers *et al*.^47^. The presence of the *VP1*-shRNA gene was confirmed by PCR amplification with forward primer 5′-ATTACCGGTAGATCCAGTTTGGTTAGTA -CCGGG-3′ and reverse primer 5′-GCTTGGAACCCTTAATATAACTTC-3′, and PCR amplification was used to generate a specific digoxigenin-labeled probe (Roche Diagnostics, Mannheim, Germany). The PCR cycles were as follows: 94 °C for 5 min; 32 cycles at 94 °C for 30 s, 52 °C for 30 s, and 72 °C for 30s; and 72 °C for 10 min. The PCR system (10 μL) contained 5 μL of 2 × ES Taq MasterMix, 1 μL of DNA, 3 μL of ddH_2_O, and 0.25 μM forward primer and reverse primer, respectively. The PCR products (5 μL) were electrophoresed in a 1% agarose gel. In the IR/DR-U6-*VP1*-shRNA + *SB100* × group, the probes were prepared by PCR amplification using the primers (Forward primer 5′-ATCCGACGCCGCCATCTC-3′; Reverse primer 5′-ACAATAGTTTTGGCAAGTCAGTT-3′) with the DIG-High-labeled substrate and cut by *XbaI* and *HpaI*. In the U6-*VP1*-shRNA and the linearized pLL3.7-*VP1*-shRNA plasmid groups, the primers and cutting sites for the labeled probe were Forward primer 5′-AAACAGCACAA -AAGGAAACTCACCC-3′, Reverse primer 5′-GATACCGTCGACCTCGAGAAAG-3 ′, *HpaI* and *XhoI*. The sheep genome DNA and the plasmid were digested with the restriction enzyme followed by electrophoresis, transfer blotting, pre-hybridization and hybridization with the labeled probe, and phosphorimager exposure. By analyzing the developed blots of Southern blotting to calculate the transgene copy numbers.

### Detection of inhibition efficiency in ear fibroblast cells from transgenic sheep against the VP1 gene

Fibroblast cells of eight positively *VP1*-shRNA integrated sheep and eight wild type sheep were harvested at the logarithmic growth phase and cultured in Dulbecco’s modified Eagle’s medium-F12 (DMEM-F12; Invitrogen, USA) supplemented with 10% fetal bovine serum. 2 × 10^5^ cells per well were seeded in a 12-well plate and cultured in fresh medium without antibiotics to achieve 80–90% confluency on the day of transfection, and psiCheck2-*VP1* was then transfected into the fibroblast cells by Lipofectamine 2000. All transfection experiments were performed in triplicate. The activities of firefly luciferase and renilla luciferase were detected by a Dual-Glo Luciferase Assay System at 48 h post transfection, and the inhibition rate was calculated according to the manufacturer’s protocol.

### Statistical analysis

All statistical analyses were performed using the IBM SPSS 22.0 software package (IBM corp., NY, USA, 2013). One-way ANOVA was used to determine significant differences followed by a multiple pair-wise comparison (Duncan’s test). The results are expressed as the means ± S.E.M., and *P* values < 0.05 were considered statistically significant.

## References

[1] Balinda SN (2009). Prevalence Estimates of Antibodies Towards Foot-and-Mouth Disease Virus in Small Ruminants in Uganda. Transboundary and Emerging Diseases.

[2] Parry NR, Barnett PV, Ouldridge EJ, Rowlands DJ, Brown F (1989). Neutralizing Epitopes of Type O Foot-and-Mouth Disease Virus. II. Mapping Three Conformational Sites with Synthetic Peptide Reagents. Journal of General Virology.

[3] Botner A (2011). Capsid proteins from field strains of foot-and-mouth disease virus confer a pathogenic phenotype in cattle on an attenuated, cell-culture-adapted virus. Journal of General Virology.

[4] Kristensen T (2016). Determinants of the VP1/2A junction cleavage by the 3C protease in foot-and-mouth disease virus infected cells. Journal of General Virology.

[5] Waterhouse PM, Wang M, Lough T (2001). Gene silencing as an adaptive defence against viruses. Nature.

[6] Chen W (2004). RNA Interference Targeting VP1 Inhibits Foot-and-Mouth Disease Virus Replication in BHK-21 Cells and Suckling Mice. Journal of Virology.

[7] Yi R (2003). Exportin-5 mediates the nuclear export of pre-microRNAs and short hairpin RNAs. Genes & Development.

[8] Zhang X (2015). Evaluation of a combinatorial RNAi lentivirus vector targeting foot-and-mouth disease virus *in vitro* and *in vivo*. Molecular Medicine Reports.

[9] Cullen BR (2005). RNAi the natural way. Nature Genetics.

[10] Vlassov AV (2007). shRNAs Targeting Hepatitis C: Effects of Sequence and Structural Features, and Comparision with siRNA. Oligonucleotides.

[11] ter Brake O, Konstantinova P, Ceylan M, Berkhout B (2006). Silencing of HIV-1 with RNA Interference: a Multiple shRNA Approach. Molecular Therapy.

[12] Mátés L (2009). Molecular evolution of a novel hyperactive Sleeping Beauty transposase enables robust stable gene transfer in vertebrates. Nature Genetics.

[13] Ivics Z (2014). Germline transgenesis in pigs by cytoplasmic microinjection of Sleeping Beauty transposons. Nature Protocols.

[14] Li W (2015). Tongue Epithelium Cells from shRNA Mediated Transgenic Goat Show High Resistance to Foot and Mouth Disease Virus. Scientific Reports.

[15] Alexandersen S, Zhang Z, Donaldson AI, Garland AJM (2003). The Pathogenesis and Diagnosis of Foot-and-Mouth Disease. Journal of Comparative Pathology.

[16] Duque H (2016). Protection induced by a commercial bivalent vaccine against Foot-and-Mouth Disease 2010 field virus from Ecuador. Vaccine.

[17] Robinson L (2011). Foot-and-Mouth Disease Virus Exhibits an Altered Tropism in the Presence of Specific Immunoglobulins, Enabling Productive Infection and Killing of Dendritic Cells. Journal of Virology.

[18] Hu S (2015). Transgenic shRNA pigs reduce susceptibility to foot and mouth disease virus infection. eLife.

[19] Robinson L (2016). Global Foot-and-Mouth Disease Research Update and Gap Analysis: 5 - Biotherapeutics and Disinfectants. Transboundary and Emerging Diseases.

[20] Wang H (2012). Identification of Short Hairpin RNA Targeting Foot-And-Mouth Disease Virus with Transgenic Bovine Fetal Epithelium Cells. PLoS ONE.

[21] de Los Santos T, Wu Q, de Avila Botton S, Grubman MJ (2005). Short hairpin RNA targeted to the highly conserved 2B nonstructural protein coding region inhibits replication of multiple serotypes of foot-and-mouth disease virus. Virology.

[22] Cong W (2010). AttenuatedSalmonella choleraesuis-mediated RNAi targeted to conserved regions against foot-and-mouth disease virus in guinea pigs and swine. Veterinary Research.

[23] Chen W (2006). Adenovirus-Mediated RNA Interference against Foot-and-Mouth Disease Virus Infection both *In Vitro* and *In Vivo*. Journal of Virology.

[24] Cong W (2010). Construction of a multiple targeting RNAi plasmid that inhibits target gene expression and FMDV replication in BHK-21 cells and suckling mice. Veterinary Research Communications.

[25] Lv K (2009). Transient inhibition of foot-and-mouth disease virus replication by siRNAs silencing VP1 protein coding region. Research in Veterinary Science.

[26] Wang H (2013). Bovine fetal epithelium cells expressing shRNA targeting viral VP1 gene resisted against foot-and-mouth disease virus. Virology.

[27] Ivics Z (2009). Transposon-mediated genome manipulation in vertebrates. Nature Methods.

[28] Horie K (2003). Characterization of Sleeping Beauty Transposition and Its Application to Genetic Screening in Mice. Molecular and Cellular Biology.

[29] Ivics Z, Izsvák Z (2010). The expanding universe of transposon technologies for gene and cell engineering. Mobile DNA.

[30] Kaufman CD, Izsvak Z, Katzer A, Ivics Z (2005). Frog Prince transposon-based RNAi vectors mediate efficient gene knockdown in human cells. J RNAi Gene Silencing.

[31] Dupuy AJ (2002). Mammalian germ-line transgenesis by transposition. Proceedings of the National Academy of Sciences.

[32] Heggestad AD, Notterpek L, Fletcher BS (2004). Transposon-based RNAi delivery system for generating knockdown cell lines. Biochemical and Biophysical Research Communications.

[33] Rumpold H (2005). RNAi-mediated knockdown of P-glycoprotein using a transposon-based vector system durably restores imatinib sensitivity in imatinib-resistant CML cell lines. Experimental Hematology.

[34] Tamhane M, Akkina R (2008). Stable gene transfer of CCR5 and CXCR4 siRNAs by sleeping beauty transposon system to confer HIV-1 resistance. AIDS Research and Therapy.

[35] Chen ZJ, Kren BT, Wong PYP, Low WC, Steer CJ (2005). Sleeping Beauty-mediated down-regulation of huntingtin expression by RNA interference. Biochemical and Biophysical Research Communications.

[36] Garrels W (2016). One-step Multiplex Transgenesis via Sleeping Beauty Transposition in Cattle. Sci Rep.

[37] Abrusán G (2016). Structural Determinants of Sleeping Beauty Transposase Activity. Molecular Therapy.

[38] Izsvák Z, Ivics Z, Plasterk RH (2000). Sleeping Beauty, a wide host-range transposon vector for genetic transformation in vertebrates. Journal of Molecular Biology.

[39] Yant SR (2000). Somatic integration and long-term transgene expression in normal and haemophilic mice using a DNA transposon system. Nature Genetics.

[40] Geurts AM (2003). Gene transfer into genomes of human cells by the sleeping beauty transposon system. Molecular Therapy.

[41] Baldassarre H (2003). Production of transgenic goats by pronuclear microinjection of *in vitro* produced zygotes derived from oocytes recovered by laparoscopy. Theriogenology.

[42] Freitas VJF (2014). The comparison of two embryo donor breeds for the generation of transgenic goats by DNA pronuclear microinjection. Animal Production Science.

[43] Menchaca A, Vilariño M, Crispo M, de Castro T, Rubianes E (2010). New approaches to superovulation and embryo transfer in small ruminants. Reproduction, Fertility and Development.

[44] Vicente JF, Freitas LMMD, Irina A, Serova LEAA (2016). The Use of Reproductive Technologies to Produce Transgenic Goats. Insights from Animal Reproduction.

[45] Li Y (2016). Efficient production of pronuclear embryos in breeding and nonbreeding season for generating transgenic sheep overexpressing TLR4. Journal of Animal Science and Biotechnology.

[46] Cognie Y, Baril G (2002). State of the art in sheep-goat embryo transfer. PRODUCTIONS ANIMALES.

[47] Hübers A (2014). Polymerase chain reaction and Southern blot-based analysis of the C9orf72 hexanucleotide repeat in different motor neuron diseases. Neurobiology of Aging.

